# Nocturnal Hypoxia in ALS Is Related to Cognitive Dysfunction and Can Occur as Clusters of Desaturations

**DOI:** 10.1371/journal.pone.0075324

**Published:** 2013-09-18

**Authors:** Su-Yeon Park, Sung-Min Kim, Jung-Joon Sung, Kyung-Min Lee, Kyung-Seok Park, Sang-Yun Kim, Hyun-woo Nam, Kwang-Woo Lee

**Affiliations:** 1 Department of Neurology, Korea Cancer Center Hospital, Seoul, Korea; 2 Department of Neurology, Seoul National University Hospital, Seoul, Korea; 3 Department of Neurology, Seoul National University, Bundang Hospital, Gyeonggi, Korea; 4 Department of Neurology, Boramae Hospital, Seoul, Korea; Central Queensland University, Australia

## Abstract

**Background:**

Amyotrophic lateral sclerosis (ALS) is a neurodegenerative disease that leads to progressive weakness of the respiratory and limb muscles. Consequently, most patients with ALS exhibit progressive hypoventilation, which worsens during sleep. The aim of this study was to evaluate the relationship between nocturnal hypoxia and cognitive dysfunction and to assess the pattern of nocturnal hypoxia in patients with ALS.

**Method:**

Twenty-five patients with definite or probable ALS underwent neuropsychologic testing, nocturnal pulse oximetry, and capnography. Patients were grouped according to the presence of nocturnal hypoxia (SpO_2_<95% for ≥10% of the night) and their clinical characteristics and cognitive function were compared.

**Results:**

Compared to patients without nocturnal hypoxia, those with nocturnal hypoxia (n = 10, 40%) had poor memory retention (p = 0.039) and retrieval efficiency (p = 0.045). A cluster-of-desaturation pattern was identified in 7 patients (70%) in the Hypoxia Group.

**Conclusions:**

These results suggest that nocturnal hypoxia can be related to cognitive dysfunction in ALS. In addition, a considerable number of patients with ALS may be exposed to repeated episodes of deoxygenation–reoxygenation (a cluster-of-desaturation pattern) during sleep, which could be associated with the generation of reactive oxygen species. Further studies are required to define the exact causal relationships between these phenomena, the exact manifestations of nocturnal cluster-of-desaturation patterns, and the effect of clusters of desaturation on ALS progression.

## Introduction

Amyotrophic lateral sclerosis (ALS) is a progressive neurodegenerative disease that involves motor neurons and leads to progressive muscle weakness [1], [2]. Weakness of the respiratory muscles in patients with ALS causes hypoventilation, which can worsen during sleep due to a weak diaphragm, sleep-disordered breathing, supine positioning, and dysfunction of the central respiratory drive [3]–[5]. A considerable number (up to 50%) of patients with ALS can also develop cognitive dysfunction involving frontotemporal lobe functions [6].

Some of the frontotemporal dysfunction in ALS may be attributable to nocturnal hypoxia in patients with ALS. A previous study showed that non-invasive positive pressure ventilation treatment partially improved cognitive function in patients with ALS [3]. In addition, another study reported that the patterns of cognitive dysfunction in patients with sleep-disordered breathing were characterized by dysfunction in the frontotemporal lobe [7], which resembles the cognitive dysfunction observed in ALS [8].

The purpose of this study was to investigate the relationship between nocturnal hypoxia and cognitive dysfunction and to assess pattern of hypoxia in patients with ALS using nocturnal continuous oximetry and capnography.

## Materials and Methods

### Patients

Patients with ALS were recruited from the ALS clinic of Seoul National University Hospital between March 2006 and July 2012. Twenty-five patients (9 women, 16 men; age range: 38–82 y) with definite or probable ALS, based on the El Escorial World Federation of Neurology Criteria were included [9]. In addition, the included patients had subjective clinical symptoms of hypoventilation (i.e., dyspnea, orthopnea, daytime drowsiness, and not feeling refreshed after sleep). Patients who were on a ventilator, required oxygen, had a tracheostomy, or had pulmonary disease were excluded. Written informed consent was obtained from all patients prior to participation. This study was approved by the Institutional Review Board of Seoul National University Hospital.

### Measurements

Capnography and pulse oximetry (CO_2_SMO, Philips Healthcare, Amsterdam, Netherlands) were used for continuous overnight respiratory monitoring. The gross respiratory pattern, average end tidal carbon dioxide (ETCO_2_) level, average oxygen saturation (SpO_2_) level, duration of nocturnal hypercapnia (percentage of sleep time when ETCO_2_>47 mmHg per total sleep time), and duration of nocturnal hypoxia (percentage of total sleep time when SpO_2_<95%) were measured and analyzed using NovaCARD software (Philips Healthcare). These measurements were chosen because the ETCO_2_ value obtained using capnography can be used to reliably reflect the partial pressure of CO_2_ in arterial blood (PaCO_2_) in patients that do not have dead space in the lungs or are not on a non-invasive ventilator and because SpO_2_ measured using pulse oximetry primarily reflects the partial pressure of O_2_ in arterial blood (PaO_2_) [5].

Respiratory patterns that reflected desaturation were also evaluated. Desaturation was defined as a ≥4% decrease in SpO_2_ and a cluster of desaturation was defined as ≥5 desaturations occurring within a 10-min period, based on a previous study [10]. Forced vital capacity (FVC) was also measured. The ALS Functional Rating Scale-Revised (ALSFRSr) was used to evaluate symptoms of orthopnea and dyspnea, degree of swallowing, speech, and salivation [11].

The Rey-Kim memory test, frontal assessment battery (FAB), verbal fluency test, and the Korean version of the mini-mental state examination (K-MMSE) were used to evaluate cognitive function. The Rey-Kim memory test is the first standardized Korean version of the auditory verbal learning test (K-AVLT) and complex figure test (K-CFT), which are widely accepted as useful tools for evaluating memory function worldwide [12]–[14]. The K-AVLT consists of 15 nouns that were read to each patient in 5 successive trials (Trials 1–5), followed by free recall, delayed recall, and delayed recognition [12], [14]. The K-CFT is used to evaluate nonverbal memory and visuospatial function by asking the patient to draw a Rey-complex figure, followed by immediate and delayed recall [13], [14]. In order to analyze the specific memory processes of registration, retention, and retrieval, the 3 components, learning curve, memory retention, and retrieval efficiency were derived from the raw scores of the Rey-Kim memory test [14]. The FAB is a short and sensitive tool for evaluating frontal lobe function [15]. Each parameter of the FAB (i.e., similarities, lexical fluency, motor series, conflicting instructions, inhibitory control, and prehension behavior) was assessed [15]. The verbal fluency test was composed of 3 components: (1) a written verbal fluency test (for Korean words beginning with the sounds for “b” and “z”), (2) a spoken verbal fluency test (for Korean words beginning with the sounds for “s” and “k”), and (3) a written categorical verbal fluency test (for animals, foods, flowers, and objects in a supermarket) [4], [8]. Patients are expected to write or say as many words as possible during the time allotted for each fluency test. The fluency index (fi, the mean time for intrinsic response generation) was then calculated for each fluency test [8], using the following equation: fi = (time allowed − time taken to read out or copy all words generated)/total number of items generated [4], [8].

### Statistical Analysis

Nocturnal hypoxia was defined as SpO_2_<95% for at least 10% of the night [16], [17]. To analyze the association between nocturnal hypoxia and cognitive function, the patients were grouped on the basis of whether they did or did not have nocturnal hypoxia. These 2 groups were analyzed and their respiratory and cognitive functions were compared using a paired *t*-test. The threshold for statistical significance was set at p<0.05. A computer software package, the Statistical Package for the Social Sciences (SPSS version 18.0 for Windows, IBM Corp., Armonk, NY USA), was used for statistical analyses.

## Results

**Figure 1:**
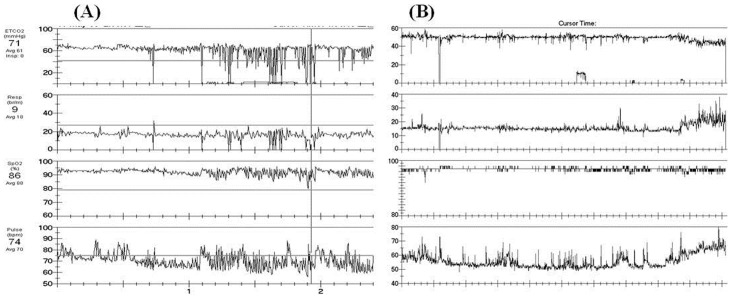
Capnography recording for patients with ALS. (A) Capnography recording showing intermittent desaturation and (B) no intermittent desaturation. Illustrated in descending order: ETCO_2_ (Top), respiratory rate, SpO_2_, and pulse (Bottom).

The respiratory and neuropsychologic characteristics of the patients are summarized in Tables 1 and 2. Ten of the 25 patients with ALS (40.0%) showed nocturnal hypoxia during capnography monitoring. A respiratory pattern with an intermittent cluster of desaturation was observed in 9 patients (Hypoxia Group: 7/10 patients [70%]; Non-Hypoxia Group: 2/15 patients [13.3%]; Figure 1). Compared to patients without nocturnal hypoxia, patients with nocturnal hypoxia showed a significantly longer duration of nocturnal hypercapnia (Hypoxia Group: 37.7±41.37; Non-Hypoxia Group: 1.86±3.92, p = 0.023), lower mean SpO_2_ (Hypoxia Group: 92.35±3.50; Non-Hypoxia Group: 96.06±0.27, p = 0.009), higher mean ETCO_2_ (Hypoxia Group: 46.92±10.36; Non-Hypoxia Group: 38.64±4.06, p = 0.036), and poorer scores for memory retention (Hypoxia Group:20.78±12.94; Non-Hypoxia Group: 49.00±36.00, p = 0.039) and retrieval efficiency (Hypoxia Group: 27.80±18.83; Non-Hypoxia Group:54.36±33.31, p = 0.045). We also found a significant correlation between duration of nocturnal hypoxia and retrieval efficiency (rho = −0.458, p = 0.049) (Figure S1). There were no significant differences between the 2 groups for age, total ALSFRSr score, duration of disease, or FVC.

**Table 1:** Clinical and respiratory characteristics of the patients with ALS.

	Nocturnal*Hypoxia Group	Nocturnal Non-Hypoxia Group	p-value
Sex (M:F)	7:3	9:6	0.627
Age (y)	51.8±10.55	56.87±12.91	0.313
Duration of disease (months)	26.2±12.72	25.87±28.86	0.973
FVC (% normal)	69.4±27.42	64.13±23.63	0.613
ALSFRSr			
Dyspnea	2.30±1.25	3.07±0.92	0.095
Orthopnea	3.10±0.74	3.36±0.50	0.318
Speech	3.20±0.42	2.73±1.22	0.190
Salivation	3.60±0.70	3.00±1.25	0.076
Swallowing	3.60±0.52	3.07±0.80	0.183
Total score	32.5±9.31	33.93±8.38	0.692
% of sleep time when ETCO_2_>47 mmHg	37.7±41.37	1.86±3.92	**0.023**
% of sleep time when SpO_2_<95%	57.10±28.95	4.71±2.98	**0.000**
Average ETCO_2_ (mmHg)	46.92±10.36	38.64±4.06	**0.036**
Average SpO_2_ (%)	92.35±3.50	96.06±0.27	**0.009**
Cluster of desaturation (n)	7 (70%)	2 (13.3%)	**0.002**

* Defined as the presence of a hypoxic period (SpO_2_<95%) for >10% of the total sleep time.

Abbreviation: ALSFRSr = Amyotrophic Lateral Sclerosis Functional Rating Scale Revised, ETCO_2_ = end-tidal carbon dioxide, FVC = forced vital capacity, SpO_2_ = arterial oxygen saturation measured using pulse oximetry, cluster of desaturation ≥5 desaturations (a decrease in SpO_2_≥4%) within a 10-min period.

All continuous values are expressed as mean ± standard deviation.

**Table 2:** Neuropsychologic characteristics of the patients with ALS.

			Nocturnal *Hypoxia Group	Nocturnal Non-Hypoxia Group	p-value
MMSE			25.6±3.63	25.62±4.13	0.993
RAVLT					
	Learning trial				
		1	4.10±1.45	4.60±2.32	0.570
		2	6.90±2.33	6.00±2.31	0.397
		3	7.40±1.78	7.10±3.03	0.790
		4	8.10±1.91	8.30±3.43	0.874
		5	9.50±2.55	9.20±3.19	0.819
	Learning curve		36.3±36.68	34.55±29.26	0.907
	**Memory retention**		**20.78±12.94**	**49.00±36.00**	**0.039**
	**Retrieval efficiency**		**27.80±18.83**	**54.36±33.31**	**0.045**
Verbal fluency					
	Spoken verbal fluency s		7.89±2.85	8.57±3.64	0.680
	Index (s)		7.07±2.79	7.21±4.06	0.953
	Spoken verbal fluency k		8.67±2.12	6.71±2.93	0.143
	Index (k)		5.71±1.28	7.96±2.44	0.098
	Written verbal fluency b		7.50±2.59	6.33±3.20	0.504
	Index (b)		6.14±3.91	8.47±3.89	0.326
	Written verbal fluency z		7.83±2.40	6.5±3.73	0.478
	Index (z)		5.68±3.06	8.27±5.57	0.342
	Category fluency: animals		11.80±4.02	10.25±4.03	0.429
	Index (animal)		5.07±1.99	5.07±1.39	0.996
	Category fluency: foods		10.67±3.28	7.38±4.57	0.105
	Index (food)		5.86±2.54	7.40±5.49	0.547
	Category fluency: flowers		8.00±3.27	6.83±3.06	0.522
	Index (flower)		5.23±5.67	5.39±4.04	0.956
	Category fluency: objects in a supermarket		8.43±3.26	8.17±3.49	0.891
	Index (supermarket)		5.52±4.68	5.13±4.36	0.877
FAB			13.8±4.71	14.33±4.72	0.794

* Defined as the presence of a hypoxic period (SpO_2_<95%) for >10% of the total sleep time.

Abbreviation: MMSE = Mini Mental State Examination, RAVLT = Rey Auditory Verbal Learning Test, FAB = frontal assessment battery.

All continuous values are expressed as mean ± standard deviation.

## Discussion

This study showed that in patients with ALS, nocturnal hypoxia was related to cognitive dysfunction and a pattern of clustered desaturations can frequently be found in patients with ALS who have nocturnal hypoxia.

To our knowledge, this is the first study to demonstrate a relationship between nocturnal hypoxia and cognitive dysfunction in ALS. Compared to ALS patients without nocturnal hypoxia, the patients with ALS and nocturnal hypoxia had significantly lower scores for memory retention and retrieval efficiency on the Rey-Kim memory test. Also, duration of nocturnal hypoxia showed significant correlation with retrieval efficiency. Frontotemporal dysfunction is a common form of cognitive impairment in patients with ALS [6], [8], [18]. Previous studies have shown that cognitive dysfunction in ALS is related to reduced vital capacity [4], implying that hypoxia or hypoventilation in these patients might, at least partially, contribute to cognitive dysfunction. The results of this study demonstrate a relationship between nocturnal hypoxia and cognitive dysfunction in ALS. These results are consistent with those of previous studies reporting reversibility of some cognitive dysfunction with non-invasive ventilation (NIV) treatment and with studies that showed a relationship between reduced vital capacity and cognitive dysfunction in ALS [3], [4]. These findings may provide an early indication for NIV treatment in patients with ALS and/or nocturnal hypoxia [3], .

In this study, 70% of the patients with ALS and nocturnal hypoxia showed a cluster-of-desaturation pattern. This finding suggests that patients with ALS are exposed to repeated episodes of abrupt deoxygenation and reoxygenation. Abrupt reoxygenation following deoxygenation has been previously shown to generate more reactive oxygen species than a simple episode of deoxygenation [19]. Taken together with previous findings demonstrating that the generation of reactive oxygen species is involved in the pathogenesis of ALS [20], our finding suggests that this cluster-of-desaturation pattern might be involved in ALS disease progression.

Though numerous studies have suggested the possibility that hypoxia might be involved in ALS disease progression, no study has shown an aggravation of the disease course with hypoxia in an animal model of ALS [21]–[23]. The current finding that a cluster of desaturation was the predominant pattern of hypoxia in patients with ALS may account for limitations seen in previous experimental studies that have attempted to evaluate the effect of hypoxia on ALS disease progression in animal models. Although intermittent hypoxia (as a cluster of desaturation) is frequently found in ALS, all previous studies have adopted a model of continuous hypoxia. These 2 types of hypoxia (intermittent or continuous) induce cell damage via discrete pathways. For example, intermittent hypoxia primarily leads to the activation of the nuclear factor kappa-light-chain-enhancer of activated B cells (NF-κB) inflammatory pathway, while continuous hypoxia primarily activates hypoxia-inducible factor (HIF) and vascular endothelial growth factor (VEGF) [24], [25]. Further studies in animal models of ALS are needed to define the exact effects of intermittent hypoxia on ALS progression.

In ALS, episodic desaturations during rapid eye movement sleep or periodic respiratory patterns consisting of mild O_2_ desaturation (i.e., sleep-disordered breathing) have been documented using polysomnography [26], [27]. In this study, the cluster-of-desaturation pattern, which is similar to that observed in previous studies, was observed in majority of ALS patients with hypoxia and also observed more frequently in the ALS-hypoxia group than in the non-hypoxic group. Although the exact mechanism remains unclear, intermittent hypoxia may be caused by neurodegenerative changes resulting in dysregulation of the respiratory responses [27].

The results of this study showed that compared to patients without nocturnal hypoxia, patients with nocturnal hypoxia had significantly lower mean SpO_2_ values, higher mean ETCO_2_ values, and a longer duration of nocturnal hypercapnia. However, there were no significant differences between the 2 groups for duration of disease, age, total ALSFRSr scores and FVC. Based on these findings, it is likely that the degree of respiratory muscle weakness and disease severity was similar in both groups. Therefore, nocturnal hypoxia may be an independent factor that reflects respiratory function in patients with ALS.

Although this study provides important findings, its results must be viewed in light of its limitations. This study was retrospective and included a relatively small sample size, which may have limited the ability to detect significant differences between groups for some tested components. In addition, although a relationship between nocturnal hypoxia and cognitive dysfunction was demonstrated, the causality of this relationship cannot be evaluated using this cross-sectional study design. Additional limitations include that polysomnography was not performed in this study to assess sleep-disordered breathing, which limited the ability to assess the detailed sleep patterns during the cluster-of-desaturation periods. Despite the potential utility of the FAB and MMSE in ALS, the FAB results did not show significant impairment in the Hypoxia Group. However, FAB results may be limited by limb muscle weakness in patients with ALS that could interfere with or even prevent patients from performing some of the tasks included in the FAB. Similarly, the MMSE scores did not differ between the groups, but it is well known that the MMSE might not be a sufficiently sensitive tool for evaluation of frontotemporal dysfunction [28].

Despite these potential limitations, this study showed that nocturnal hypoxia in patients with ALS is related to cognitive dysfunction and that nocturnal hypoxia in ALS is primarily characterized by a cluster-of-desaturation pattern that is associated with increased risk of reactive oxygen species generation responses [19], [20]. Further studies are required to define the exact causal relationships between these phenomena, the exact manifestations of nocturnal cluster-of-desaturation patterns, and the effect of clusters of desaturation on disease progression in ALS.

## Supporting Information

Figure S1 **The retrieval efficiency in ALS patients correlated significantly with duration of nocturnal hypoxia (Spearman correlation analysis, rho = −0.458, p = 0.049).**
(TIF)
