# Supplementary figures and images for: Nocturnal Hypoxia in ALS Is Related to Cognitive Dysfunction and Can Occur as Clusters of Desaturations
Source: PLoS One. 2013 Sep 18;8(9):e75324. doi: 10.1371/journal.pone.0075324 (PMC3776791; doi:10.1371/journal.pone.0075324)

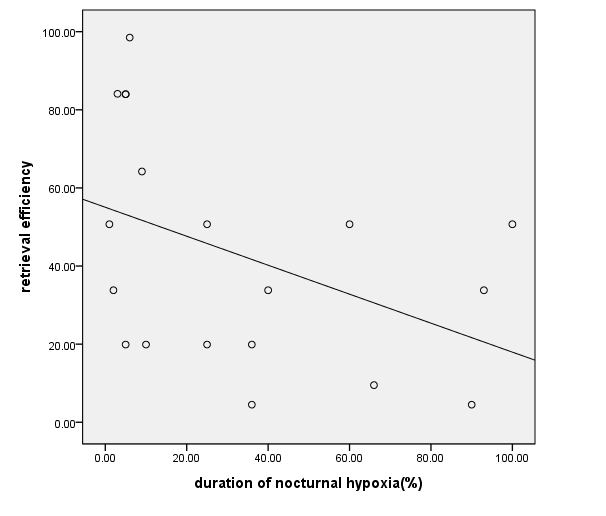

Supplement: Figure S1 — The retrieval efficiency in ALS patients correlated significantly with duration of nocturnal hypoxia (Spearman correlation analysis, rho = −0.458, p = 0.049). (TIF) [file pone.0075324.s001.tif]
